# Inanition in Carcinoma

**Published:** 1916-02

**Authors:** Arnold H. May


### Inanition in Carcinoma.

ARNOLD H. MAY, M. D.

In cases of carcinoma of the stomach, particularly in those cases involving the cardiac and pyloric orifices, the question of nutrition is one of primary importance. Sooner or later, this is the predominating factor in these cases, and inanition is often the determining cause of the end long before the malignant process. Whereas in these cases of carcinoma there exists a marked cachexia, the condition of inanition resulting from inadequate feeding is often overlooked, or considered too late, hence patients pass into a condition of exhaustion and collapse. Knowing the fatality of the disease, those attending the sick often consider this mechanic interference to nutrition in no special light, since it simply hastens the end to a miserable existence. Nevertheless, the fact remains that death resulting from inanition is in a sense premature, since in many cases life may be prolonged if these measures designed to overcome inanition are employed, and during this period there results also a palliation of symptoms. Too often, however, in spite of all, inanition must be the determining factor of the end.

The measures employed to maintain the patient and supply him with adequate heat units are well known to the profession. In malignancy of the oesophagus and cardia before there is complete stricture, a liquid diet rich in foods of high caloric value is indicated. Here the necessary calories are introduced in milk, cream, eggs finely beaten, butter strained, and highly nutritious broths, oils, and perhaps scraped or chopped meats, artificial albumens; and in fact any liquid or liquifiable, highly nutritious, digestible food is indicated. Not only variety, but adequate amount is essential, the dietary containing at least 2,000 heat units per 24 hours. Cohnheim's dietary is as follows:

8 a. m.—Tea with 125 gms. of cream;
9 a. m.—Milk (250 gms.)
11 a. m.—Flour soup with 125 gms. of cream and butter;
1 p. m.—Bouillon with a tablespoonful of flour and one or two yolks of eggs and butter;
4 p. m.—Tea with 125 gms. of cream;
6 p. m.—Flour soup or milk;
8 p. m.—Bouillon with rice or flour and butter.

With advancing cardiac stenesis the above diet will be found soon to be impossible—foods being regurgitated into the mouth. When this occurs an attempt may be made to dilate the stricture by means of sounds—the employment of hollow sounds being considered. The use of sounds demands caution, since great irritation or even perforation may result. As a rule, however, natural feeding being impossible, an inadequate amount of food reaching the stomach, the physician must decide quickly between gastrostomy, or inanition and subsequent death. The earlier as a rule gastrostomy is determined upon (and no special contraindication existing, e. g. extreme cachexia, etc.) the better will be chance of prolongation of life. It has oftentimes been the custom at this stage to employ and rely upon rectal feeding. An assimilation of predigested food by the colon is possible, as is also the absorption of a large variety of nutrient material. Effective digestion of food value of molecules of solid undigested food per rectum is very small. The colon absorbs fluids; enemata thus increasing the fluids of the body. As an adjuvant method of nutrition, or employed over short periods, rectal feeding has a distinct value, but relied upon alone inanition results. Gastrostomy being performed feeding can proceed through the fistula aud per rectum.

In cases of malignant stenosis of the pylorus the first indication is to give suffiicient food of a highly nutritious character in the liquid state, and in small quantities, so as to permit of its expulsion into the duodenum. The stenotic pylorus causes hypertrophy, and later atony of the stomach muscle, so that retention and stagnation occur. The mucous membrane undergoes changes, and atrophy, so that hydrochloric acid is absent or diminished, and hence the food undergoes putrefactive and fermentative changes, the small amount reaching the duodenum being diminished in food value or even toxic. Nutrition quickly suffers under these circumstances. A measure then designed to permit of the passage of food from the stomach to intestines is indicated, and gastroenterostomy is the operation of choice.

Gastroenterostomy theoretically ideal is practically questionable, since the mortality and sequelae of it in the hands of those not especially skilled often make it prohibitive. Gastroenterostomy successfully performed readily permits of feeding per os. Rectal enemata may also be used as an adjuvant measure. The average prolongation of life after gastroenterostomy is according to Krönlein ''193 days.'' A palliation of many symptoms also following the operation.

If in these cases having considered the mechanical impediment to ingestion of food, inanition is early combatted, the life of the patient may be prolonged. No successful remedy having been discovered to combat visceral carcinoma (except surgery early) the physician must content himself with the employment of those measures which at most prolong life or palliate symptoms.

